# A Randomized Controlled Trial of Thermo-Sensitive Sol–Gel Anti-Adhesion Agent after Gynecologic Surgery

**DOI:** 10.3390/jcm9072261

**Published:** 2020-07-16

**Authors:** Young Im Kim, Maria Lee, Se Ik Kim, Aeran Seol, Eun Ji Lee, Hee Seung Kim, Yong Sang Song

**Affiliations:** 1 Department of Obstetrics and Gynecology, Seoul National University Hospital, Seoul 03080, Korea; milka.kim@gmail.com; 2 Department of Obstetrics and Gynecology, Seoul National University College of Medicine, Seoul 03080, Korea or marialee@snu.ac.kr (M.L.); seikky@naver.com (S.I.K.); sar314@naver.com (A.S.); bliss880103@gmail.com (E.J.L.); bboddi0311@gmail.com (H.S.K.); 3 Cancer Research Institute, Seoul National University College of Medicine, Seoul 03080, Korea

**Keywords:** abdominal adhesion, gynecologic surgery, anti-adhesion agent, thermo-sensitive agent, sol–gel process

## Abstract

Postoperative abdominal adhesions can lead to several adverse consequences such as pelvic pain, bowel obstruction, and infertility. We aimed to explore the anti-adhesion efficacy and safety of a thermo-sensitive sol–gel agent in patients who receive abdominopelvic surgery for benign gynecologic disease. This study was a randomized, controlled, single-blind clinical trial of women undergoing benign gynecologic surgery between January 2017 and December 2017. The patients were randomly assigned to three groups with a 1:1:1 ratio: experimental group (received the thermo-sensitive sol–gel agent), control group (untreated), and comparator group (received 4% icodextrin). Patients were followed for 4 weeks postoperatively, and efficacy was evaluated by performing the visceral slide test to identify adhesion formation. In total, 183 patients were enrolled in the study, and 178 (97.3%) completed the trial. The incidence rate of abdominal adhesion formation was significantly lower in the experimental group than in the control group (7.9% vs. 21.1%, *p* = 0.040); however, it was similar between the experimental and comparator groups (7.9% vs. 13.8%. *p* = 0.299). At 4 weeks, no differences in adhesion-related symptoms were observed between the experimental and control groups. Adverse events were mostly mild and did not differ significantly among the three groups (*p* = 0.375). In conclusion, use of a thermo-sensitive sol–gel agent was safe and effective to prevent abdominal adhesions after benign gynecologic surgeries.

## 1. Introduction

Abdominal adhesions develop in more than half of women following gynecologic surgery [1,2]. Referring to formation of fibrous bands between normally separated abdominal tissues and organs, abdominal adhesions can lead to several adverse consequences, such as pelvic pain, bowel obstruction, and infertility [3,4,5]. Thus, development of effective strategies minimizing postoperative adhesion formation is necessary [6].

Beside meticulous surgical technique, anti-adhesion barriers can be administered intra-operatively over the surgical site to act as a physical barrier, blocking contact with surrounding tissue and thereby reducing adhesion formation. Membrane-form agents are effective, but they are difficult to apply during laparoscopic surgery [7,8,9,10]. Liquid barriers are effective, but they tend to flow away from the surgical site before performing their anti-adhesion function. Adept (Baxter, Deerfield, IL, USA), a more advanced liquid anti-adhesion agent consisting of a 4% icodextrin solution, is known to remain in the abdominal cavity for at least 3–4 days, preventing adhesion formation by a colloid osmotic action, however, a large amount of it should be instilled [4].

Thermo-sensitive sol–gel agents may break through the shortcomings of solid and liquid barriers. Mediclore (CGBio Inc., Seongnam-si, Korea), a poloxamer-based thermo-sensitive sol–gel approved by the Korean Ministry of Food and Drug Safety in October 2014, maintains a sol state at room temperature, which allows it to be easily injected, but it transforms to a gel state at body temperature, which allows it to remain in one place. Therefore, Mediclore is expected to have a substantial anti-adhesion capability.

To explore the efficacy of applying Mediclore during abdominopelvic surgery for benign gynecologic disease to prevent adhesions, we conducted a single-center, randomized, controlled, subject-blind clinical trial comparing Mediclore to no treatment. In addition, we used Adept as an active comparator. Instead of invasive second-look surgery, we assessed adhesion formation using the visceral slide test, which is a non-invasive, easily performed method with a high prediction rate.

## 2. Materials and Methods

### 2.1. Trial Design

This is a single-center, randomized, controlled, single-blind trial to assess clinically relevant hypotheses: use of a thermo-sensitive sol–gel agent prevents intra-abdominal adhesions after benign gynecologic surgeries. This trial employed a parallel design. Using stratified block randomization, random allocation tables were prepared according to the surgical site (uterus or adnexa), with a 1:1:1 ratio for three groups: the experimental (Mediclore-treated), control (no treatment), and comparator (Adept-treated) groups. Patients who met the inclusion criteria during screening and did not exhibit any of the exclusion criteria were assigned to each group in order, using sealed envelopes. The patients were under general anesthesia when the treatment was administered and were thus unaware of their group assignment.

The study was approved by the Institutional Review Board (no. C-1608-027-783) and posted to clinicaltrials.gov (NCT03007654). Each patient provided written informed consent before any trial-specific procedures were conducted.

### 2.2. Inclusion and Exclusion Criteria

We enrolled women over 20 years of age scheduled to undergo abdominopelvic surgery for benign gynecologic diseases at Seoul National University Hospital in Korea. The exclusion criteria were as follows: (1) patients who had a history of previous surgery at the intended surgical site; (2) those who were enrolled in other clinical trials and received medical intervention within 4 weeks before registration of this study; (3) those who were diagnosed with systemic or pelvic infection within 1 week before the registration; and (4) those who underwent anticoagulation or systemic immunosuppressive treatment within 1 week before surgery. We also excluded patients who were pregnant, or had immunosuppressive or autoimmune disorder, or any serious condition that may affect the perioperative period (e.g., uncontrolled diabetes and coagulation disorder).

### 2.3. Intervention

Mediclore is a mixture of water-soluble polymeric composites contained in a single-use, prefilled, sterile 5 mL syringes. In the experimental group, 5 mL of Mediclore was applied evenly throughout the surgical site just before wound closure. Adept is a colorless, non-viscous, iso-osmolar 4% solution of icodextrin. In the comparator group, Adept was used as an intra-operative lavage at the rate of at least 100 mL every 30 min. At the end of the surgery, 1 L of Adept was instilled into the abdominopelvic cavity. In the control group, no anti-adhesion agent was applied.

### 2.4. Assessment

Efficacy and safety of the anti-adhesion agents were evaluated for a total of 4 weeks postoperatively. The patients underwent four study-related visits. Visit 1 occurred within 2 weeks before surgery and involved screening for study eligibility, consisting of a past medical/surgical history, co-morbidities and current medications, physical examination, and laboratory tests. A baseline visceral slide test was also performed at visit 1 for evaluation of preoperative abdominal adhesion. Visit 2 occurred on the day of surgery, at which time the patient was randomized to one of the three study groups. Visit 3 occurred at 1 week after surgery, at which survey for adhesion symptoms and follow-up assessments including a medication history, physical examination, and laboratory tests were conducted. Visit 4, which occurred 4 weeks postoperatively, involved evaluations of primary and secondary outcomes. Follow-up assessments were the same as those at visit 3 (Figure S1).

### 2.5. Primary Outcome: Efficacy on Abdominal Adhesion Formation

At visit 4, gynecologic surgeons performed a visceral slide test using transabdominal ultrasonography to assess abdominal adhesion formation. Visceral slide refers to the normal, spontaneous, longitudinal movement of viscera during excessive breathing motions [11]. To evaluate visceral slide, an abdominal transducer was placed in a sagittal plane at five abdominal zones: right upper quadrant (RUQ), right lower quadrant (RLQ), left upper quadrant (LUQ), left lower quadrant (LLQ), and the umbilicus. An abnormal visceral slide test or the presence of abdominal adhesions was defined as less than 1 cm movement of internal organs along the longitudinal axis during forced respiratory inspiration and expiration.

### 2.6. Secondary Outcomes: Adhesion Symptoms, Satisfaction for Patients and Surgeons, and Safety

At visits 3 and 4, the presence and severity of adhesion symptoms (e.g., loss of appetite, vomiting, and abdominal pain) were assessed using a 4-point Likert scale: 0 = none, 1 = mild, 2 = moderate, and 3 = severe. The severity of abdominal or pelvic pain was also assessed at the time of visits 1 and 4 using the continuous 100 mm visual analogue scale (VAS), ranging from 0 = no pain to 10 = worst pain imaginable. On the day of surgery, we evaluated surgeon satisfaction regarding the convenience of applying Mediclore or Adept. At visit 4, patient satisfaction and surgeon satisfaction were evaluated using a 5-point Likert scale: 0 = poor, 1 = fair, 2 = good, 3 = very good, and 4 = excellent. In terms of safety, patients received physical examination at visits 2, 3, and 4, and laboratory tests including hematology, coagulation, biochemistry, and urinalysis at visits 3 and 4. Adverse events during the trial were assessed through consultations as well as inquiries during regular or additional patient visits. For each adverse event, we recorded the date of occurrence, resolved date, severity, consequences, corresponding actions, and causality. Besides the regular study visits, additional visits were arranged if necessary for safety assessment.

### 2.7. Statistical Analysis

Based on the results of a meta-analysis study regarding adhesions after gynecologic surgery in patients receiving hyaluronan gel anti-adhesion agents or no treatment, the incidence of adhesions at 4 weeks postoperatively was assumed to be 50.0% (35/70) and 76.6% (49/64) in the treatment and control groups, respectively [4]. Setting the allocation ratio at 1:1 and two-sided significance level at 5%, the minimum number of patients required for 80% statistical power was 51 patients per group. Estimating a drop-out rate of 20%, we enrolled 64 patients in each group.

Statistical analyses were performed in three datasets: (1) the full analysis set (FAS) that included patients for whom data were available for evaluating the primary outcome; (2) the per protocol set (PPS) consisted of only those who completed the clinical trial without any major protocol violations; (3) the safety set consisted of all patients from the three groups.

The primary outcome, efficacy, was described as the percentage of patients with abnormal visceral slide test results in any of five zones assessed at visit 4 and was compared between the experimental and control groups using Pearson’s chi-square or Fisher’s exact tests. For exploratory purposes, efficacy evaluations were also conducted between experimental and comparator groups, as well as between comparator and control groups.

Differences in baseline characteristics and secondary outcomes between the two groups or among the three groups were assessed with Pearson’s chi-square or Fisher’s exact tests for categorical variables and with Student’s *t*-, Wilcoxon’s rank sum, ANOVA, or Kruskal–Wallis tests for continuous variables. All statistical analyses were performed using SAS software version 9.2 (SAS Institute Inc., Cary, NC, USA), and *p* < 0.05 was considered statistically significant.

## 3. Results

### 3.1. Study Population

**Figure 1:**
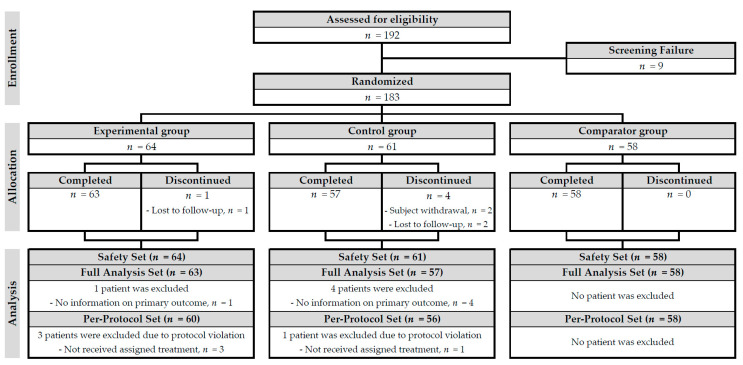
Flow diagram of the progress through the phases of a parallel randomized trial.

From January 2017 through December 2017, a total of 192 patients were screened. Among them, 9 encountered screening failure: 2 were ineligible for entry into this study and 7 were registered for the trial but did not undergo randomization. In total, 183 patients were randomly assigned to the three groups, and 178 (97.3%) completed the study. Of the 5 patients who failed to complete the study, 2 withdrew their consent and 3 were lost to follow-up. Regarding the study outcomes, primary outcomes were not reported in 1 and 4 from the experimental and control groups, respectively. Major protocol violations occurred in 3 and 1 from the experimental and control groups, respectively; they did not receive appropriate treatment according to their assigned group (Figure 1).

Thus, the FAS included 178 patients (63, 57, and 58 in the experimental, control, and comparator groups, respectively) while the PPS included 174 patients (60, 56, and 58). Safety analysis was available in 183 patients (64, 61, and 58) (Figure 1).

### 3.2. Demographics and Baseline Characteristics

Patients’ characteristics of the FAS are summarized in Table 1. Patient age, body mass index, co-morbidity, indication for the gynecologic surgery, and presence of preoperative abdominal adhesions were similar among the three groups. However, the proportion of patients who underwent laparoscopic surgery was different among the experimental, control, and comparator groups (74.6% vs. 87.7% vs. 63.8%; *p* = 0.012). In terms of surgical procedure, myomectomy was less frequently performed in the control group, compared to the experimental group (7.0% vs. 22.2%; *p* = 0.020) or comparator group (7.0% vs. 27.6%; *p* = 0.004), which might lead to the difference in mean operative time among the three groups. Surgical procedures other than myomectomy and anatomical site of surgery were similar among the groups.

### 3.3. Efficacy on Abdominal Adhesion Formation

Results for the primary outcome of the current study are presented in Table 2. The visceral slide test at visit 4 demonstrated significantly lower incidence of abdominal adhesion formation in the experimental group compared to the control group (FAS, 7.9% vs. 21.1%, *p* = 0.040; and PPS, 8.3% vs. 21.4%, *p* = 0.046). Specifically, adhesion formation in the LLQ zone was significantly less in the experimental group than in the control group (FAS, 1.6% vs. 12.3%, *p* = 0.026; and PPS, 1.7% vs. 12.5%, *p* = 0.028). In the comparative analyses for abdominal adhesion formation between the experimental and comparator groups, no significant differences were observed in total abdomen or by five different zones.

### 3.4. Adhesion Symptoms

The presence and severity of adhesion symptoms are summarized in Table 3. At visit 4, none of patients in this study reported fever or any moderate to severe specific symptoms. Between the experimental and control groups, there were no differences in loss of appetite, abdominal pain, vomiting, and abdominal distension. In the comparative analyses, the proportion of patients with abdominal pain was significantly higher in the comparator group than in the experimental group (FAS, *p* = 0.007; PPS, *p* = 0.006) or the control group (FAS, *p* = 0.005; PPS, *p* = 0.006). However, none of them reported moderate to severe abdominal pain. Other adhesion symptoms were similar between the experimental and comparator groups.

Table 4 presents changes in pain VAS scores between baseline (visit 1) and visit 4. The baseline pain VAS score was significantly lower in the experimental group compared to that in the control group (FAS, mean, 2.3 vs. 6.4, *p* = 0.007; PPS, mean, 1.8 vs. 6.5, *p* = 0.004). Although the mean change in pain VAS scores was significantly greater in the experimental group (FAS, 1.2 vs. −2.8; *p* = 0.003; PPS, 1.7 vs. −2.8, *p* = 0.002), the pain VAS score at visit 4 was similar between the two groups. In the comparative analyses, patients in the experimental and comparator groups showed both similar pain VAS scores at visit 4 and changes in pain VAS scores between visit 4 and baseline.

### 3.5. Satisfaction for Patients and Surgeons

Satisfaction results assessed by both patients and surgeons at visit 4 are presented in Table S1. Overall patient satisfaction did not differ significantly between the experimental and comparator groups. In contrast, overall surgeon satisfaction was significantly higher in the experimental group than in the comparator group (*p* < 0.001). Surgeon satisfaction with the convenience of applying the anti-adhesion agent assessed on the day of surgery was also significantly higher in the experimental group than in the comparator group (*p* < 0.001) (Table S2).

### 3.6. Safety

There were no differences among the three groups with respect to changes from baseline and to any time later in the study with regard to vital signs, hematologic and coagulation blood test results, or urine test results. In contrast, the mean change in aspartate aminotransferase (AST) between visit 3 and baseline differed significantly among the groups: 2.5, 1.6, and −0.4 in the experimental, control, and comparator groups, respectively (*p* = 0.030). However, changes in AST between visit 4 and baseline were not significantly different. There were also significant differences among the three groups with regards to changes in total cholesterol between visit 3 and baseline (10.3, 3.1, and −4.1 in the experimental, control, and comparator groups, respectively; *p* = 0.014), and between visit 4 and baseline (12.8, 10.6, and 1.4 in the experimental, control, and comparator groups, respectively; *p* = 0.039).

Comparisons of adverse events among the groups are presented in Table S3. Treatment emergent adverse events (TEAEs) were reported in 84.4% (54/64; 121 events), 77.0% (47/61; 103 events), and 86.2% (50/58; 122 events) of patients in the experimental, control, and comparator groups, respectively, without significant differences (*p* = 0.375). Most adverse events were checked mild. Three patients had serious TEAEs, which were not attributed to the interventions of the current study: one with hematochezia (experimental group), one with connective tissue disorder (control group), and one with bladder injury (comparator group). All the serious TEAEs resolved with medication or supportive care. Adverse device effect was observed in one patient (experimental group). The affected patient experienced abdominal pain, but it was mild, therefore, no specific treatment was required.

## 4. Discussion

In this prospective, randomized, controlled clinical trial involving patients undergoing gynecologic abdominopelvic surgery, use of a poloxamer-based thermo-sensitive sol–gel agent (Mediclore) was significantly associated with decreased adhesion formation, compared with no anti-adhesion treatment. In addition, Mediclore appeared to be a safe strategy for preventing postoperative adhesions.

Postoperative abdominal adhesion formation is frequent and a burden to both patients and clinicians: for example, patients might suffer from small-bowel obstruction requiring surgical interventions, and clinicians might experience increase of workload [12,13,14]. A disruption in the epithelial or mesothelial layer of tissue during surgery initiates a series of processes, such as inflammation, coagulation cascade, and angiogenesis, altogether resulting in adhesions [15,16]. Considering the mechanism underlying adhesions, the use of anti-adhesion barriers makes sense in preventing adhesion formation because it separates the surgical area from surrounding tissues and prevents attachment of fibrin clots [16,17]. Moreover, a recent study reported that the use of anti-adhesion barriers in open colorectal surgery was even cost-effective in preventing adhesion-related problems [18]. In gynecologic surgery, the guidelines from the Anti-Adhesions in Gynecology Expert Panel group recommend that surgeons should consider the use of anti-adhesion barriers as part of the adhesion reduction strategy [19]. To evaluate abdominal adhesion formation after surgery, second-look surgery is a gold standard method. However, because of its invasive nature, this method raises ethical issues and is difficult to use in real-world clinical practice. Instead, non-invasive imaging modalities might be alternative options [20]. Previously, our research team validated the diagnostic performance of the ultrasound-based visceral slide test in a prospective cohort of 144 patients scheduled to undergo benign gynecologic surgeries [11]. In that study, the visceral slide test showed a sensitivity of 97.2%, specificity of 68.6%, positive predictive value of 90.6%, negative predictive value of 88.9%, and accuracy of 90.3%, suggesting it is a simple, reliable assay for identifying intra-abdominal adhesions. In addition, the ultrasound-based visceral slide test is very easy to perform and takes only few minutes, resulting in minimal inter-observer discrepancy. Therefore, we adopted this non-invasive method to assess abdominal adhesion formation after surgery.

Recently, cine-magnetic resonance imaging (cine-MRI) was also proposed as a useful imaging modality to identify patients with adhesions [20,21]. This non-invasive modality covers the whole abdomen and can locate adhesions. In a prospective cohort study including patients with a history of abdominal surgery and chronic abdominal pain, a decision-making process based on cine-MRI was effective in triaging the patients to operative or conservative treatment [21]. Therefore, cine-MRI might be an alternative to ultrasonography in the assessment of postoperative adhesion formation. However, MRI is more expensive and takes a longer time to perform, compared to ultrasonography.

Mediclore is a product using a thermo-sensitive mechanism by poloxamer. Poloxamer consists of hydrophilic polyethylene glycol (PEG) and hydrophobic polypropylene glycol (PPG) blocks, arranged in a triblock structure PEGx–PPGy–PEGx. As temperature increases, poloxamer copolymer molecules aggregates into micelles. This micellization is due to the dehydration of hydrophobic PPG blocks, which represents the first step in the gelation process. Over time, these micelles are gradually piled up in an array to form a gel. The hydrophilic PEG block of poloxamer has mucoadhesive properties so that it can be maintained without dislocation from the applied area [22,23]. In addition to poloxamer, Mediclore contains gelatin and chitosan, which ensure stable attachment to the surgical field and have hemostatic and antibacterial effects [24,25,26]. Biocompatibilities of poloxamer, chitosan, and gelatin have been demonstrated for a long time through preclinical and clinical studies.

The anti-adhesion effect and safety of Mediclore have been proven in preclinical animal studies [27,28] as well as in clinical studies including transurethral prostate resection (TURP) [29], total knee arthroplasty [30], and parotidectomy [31]. For example, use of Mediclore during TURP in patients with prostatic hyperplasia was significantly associated with a decreased risk of urethral stricture and a higher peak urine flow rate, compared to the instillation of lubricant [29]. However, Mediclore has not yet been investigated in benign gynecologic surgeries; to our knowledge, the current study is the first in this research field.

In this study, Adept, 4% icodextrin, was set as an active comparator, and no significant difference in abdominal adhesion formation was observed between the experimental and comparator groups, suggesting similar anti-adhesion effects of the two agents. However, in order to achieve the anti-adhesion effect, at least 1 L of Adept should be administered intra-operatively [4], which may cause discomfort to patients or inconvenience to surgeons. In the current study, surgeon satisfaction with handling the agent was significantly higher for Mediclore than for Adept.

Of note, although our trial was randomized, the experimental group included a higher proportion of patients who underwent myomectomy, compared with the control group. Our results with a poloxamer-based thermo-sensitive sol–gel agent compare favorably to those of a previous study with a hyaluronic acid-based gel that reported the incidence rate of adhesions after myomectomy as 33% [3]. Moreover, patients in the experimental group received laparoscopic surgery less frequently, compared to those in the control group (74.6% vs. 87.7%). The surgical approach may influence the development of postoperative abdominal adhesions [32]. However, studies comparing the adhesion risk in laparoscopy versus that in laparotomy have given mixed results [33,34]. Despite the difference in the type of surgery between the groups in the current study, the anti-adhesion effect can be evaluated as good in the experimental group.

In the current study, there were no significant differences between the experimental and control groups when assessing adhesion symptoms. However, changes in VAS pain scores between visit 4 and baseline were greater in the experimental group than those in the control group. This result seems to be attributed to the difference in baseline pain VAS scores between the two groups. Of note, by 4 weeks after surgery, abdominal pain was either absent or mild in all patients, which was consistent with the lack of difference in overall patient satisfaction between groups at that time.

Mediclore has been previously demonstrated to be safe and effective in the prevention of adhesion formation after various surgical procedures [29,30,31]. A recent study reported that the use of another poloxamer-based thermo-sensitive anti-adhesion agent reduced the incidence of intestinal obstruction after radical gastrectomy for gastric cancer without increasing the risk of complications [35]. In line with the previous studies, we also demonstrated the adhesion-prevention effects and safety of Mediclore in patients who underwent benign gynecologic surgeries.

Our study has several limitations. First, the 4-week follow-up period was relatively short, which may not reflect the long-term efficacy of Mediclore. Although we checked patient safety and adhesion-related complications in many respects, we admit that long-term assessment for those were not conducted in this study. We are now planning further prospective studies of more delicate designs covering long-term follow-up. Second, despite the randomized controlled trial design, the proportion of laparoscopic surgery was different among the experimental, control, and comparator groups. However, subgroup analysis according to surgical approach was not performed in this study. Further prospective trials with large sample size would determine whether the effects of Mediclore differ between laparotomy and laparoscopic surgery. Lastly, although the visceral slide test is a simple, validated test that provides a high prediction rate of abdominal adhesion, further studies are needed to establish the usefulness of the test with respect to long-term outcomes. Despite these limitations, this study is meaningful, as it is the first prospective, randomized, controlled study to examine the efficacy and safety of Mediclore for abdominopelvic gynecologic surgery.

## 5. Conclusions

In conclusion, this study demonstrated that a thermo-sensitive sol–gel agent provides anti-adhesion effects without safety concerns in patients undergoing abdominopelvic surgery for benign gynecologic disease. Surgeon satisfaction with handling this agent was significantly higher compared to that with a 4% icodextrin solution.

## Figures and Tables

**Table 1:** Demographics and baseline characteristics of the patients in full analysis set.

Variables	Experimental Group (*n* = 63)	Control Group (*n* = 57)	Comparator Group (*n* = 58)	*p*
Age (year)	41.0 ± 9.1 (24.0–60.0)	42.7 ± 11.4 (21.0–75.0)	42.4 ± 9.2 (24.0–70.0)	0.607 ^1^
Height (cm)	159.8 ± 6.3 (130.0–172.0)	160.0 ± 4.5 (152.0–170.0)	159.7 ± 5.5 (148.0–170.0)	0.965 ^2^
Weight (kg)	59.0 ± 9.9 (37.0–105.0)	60.8 ± 12.7 (41.3–108.6)	59.7 ± 9.6 (45.2–89.1)	0.900 ^2^
Body Mass Index (kg/m^2^)	23.2 ± 3.9 (17.4–42.6)	23.8 ± 4.9 (15.9–41.9)	23.5 ± 3.8 (16.5–35.2)	0.929 ^2^
Co-morbidity	-	-	-	0.722 ^3^
Hypertension	4 (6.3)	6 (10.5)	4 (6.9)	0.660 ^3^
Diabetes	1 (16)	1 (1.8)	2 (3.4)	0.752 ^3^
Dyslipidemia	0	2 (3.5)	3 (5.2)	0.211 ^3^
Others	9 (14.3)	10 (17.5)	6 (10.3)	0.538 ^3^
Gynecologic disease	-	-	-	-
Uterine myoma	28 (44.4)	22 (38.6)	34 (58.6)	0.085 ^3^
Adenomyosis	2 (3.2)	5 (8.8)	2 (3.4)	0.299 ^3^
Ovarian neoplasm	34 (54.0)	35 (61.4)	29 (50.0)	0.459 ^3^
Cervical dysplasia	1 (1.6)	0	0	0.399 ^3^
Endometrial hyperplasia	1 (1.6)	1 (1.8)	1 (1.7)	0.997 ^3^
Surgical approach	-	-	-	0.012 ^3^
Laparotomy	16 (25.4)	7 (12.3)	21 (36.2)	-
Laparoscopy	47 (74.6)	50 (87.7)	37 (63.8)	-
Surgical procedure	-	-	-	-
Myomectomy	14 (22.2)	4 (7.0)	16 (27.6)	0.014 ^3^
Hysterectomy	18 (28.6)	24 (42.1)	20 (34.5)	0.298 ^3^
Ovarian cystectomy	25 (39.7)	21 (36.8)	16 (27.6)	0.351 ^3^
Salpingo-oophorectomy	10 (15.9)	18 (31.6)	14 (24.1)	0.128 ^3^
Salpingectomy	1 (1.6)	3 (5.3)	1 (1.7)	0.396 ^3^
Anatomical site of surgery	-	-	-	0.278 ^3^
Uterus only	27 (42.9)	18 (31.6)	28 (48.3)	-
Uterus + unilateral adnexa	2 (3.2)	3 (5.3)	3 (5.2)	-
Uterus + bilateral adnexa	3 (4.8)	7 (12.3)	5 (8.6)	-
Unilateral adnexa	21 (33.3)	13 (22.8)	10 (17.2)	-
Bilateral adnexa	10 (15.9)	16 (28.1)	12 (20.7)	-
Preoperative adhesion	-	-	-	0.895 ^3^
Present	23 (36.5)	19 (33.3)	19 (32.8)	-
Absent	40 (63.5)	38 (66.7)	39 (67.2)	-
Operative time (min)	85.9 ± 53.3 (25–345)	77.1 ± 42.8 (10–200)	96.2 ± 42.9 (20–205)	0.029 ^2^

Presented with mean ± SD (range) or *n* (%). ^1^ ANOVA or ^2^ Kruskal–Wallis test and ^3^ chi-squared test were used. Abbreviations: SD, standard deviation.

**Table 2:** Incidence rate of abdominal adhesion formation assessed by visceral slide test at 4 weeks after surgery.

	Experimental Group ^*, †^	Control Group ^*, ‡^	Comparator Group ^†, ‡^	^*^ *p*	^†^ *p*	^‡^ *p*
Full analysis set	(*n* = 63, %)	(*n* = 57, %)	(*n* = 58, %)	-	-	-
Total abdomen	-	-	-	0.040 ^1^	0.299 ^1^	0.305 ^1^
Abnormal	5 (7.9)	12 (21.1)	8 (13.8)	-	-	-
Normal	58 (92.1)	45 (78.9)	50 (86.2)	-	-	-
Umbilicus	-	-	-	>0.999 ^2^	>0.999 ^2^	>0.999 ^2^
Abnormal	2 (3.2)	1 (1.8)	2 (3.4)	-	-	-
Normal	61 (96.8)	56 (98.2)	56 (96.6)	-	-	-
RUQ	-	-	-	>0.999 ^2^	>0.999 ^2^	>0.999 ^2^
Abnormal	1 (1.6)	0	1 (1.7)	-	-	-
Normal	62 (98.4)	57 (100.0)	57 (98.3)	-	-	-
RLQ	-	-	-	0.255 ^2^	0.670 ^2^	0.490 ^2^
Abnormal	2 (3.2)	5 (8.8)	3 (5.2)	-	-	-
Normal	61 (96.8)	52 (91.2)	55 (94.8)	-	-	-
LUQ	-	-	-	>0.999 ^2^	>0.999 ^2^	>0.999 ^2^
Abnormal	1 (1.6)	1 (1.8)	1 (1.7)	-	-	-
Normal	62 (98.4)	56 (98.2)	57 (98.3)	-	-	-
LLQ	-	-	-	0.026 ^2^	0.054 ^2^	0.743 ^2^
Abnormal	1(1.6)	7 (12.3)	6 (10.3)	-	-	-
Normal	62 (98.4)	50 (87.7)	52 (89.7)	-	-	-
Per protocol set	(*n* = 60, %)	(*n* = 56, %)	(*n* = 58, %)	-	-	-
Total abdomen	-	-	-	0.046 ^1^	0.344 ^1^	0.284 ^1^
Abnormal	5 (8.3)	12 (21.4)	8 (13.8)	-	-	-
Normal	55 (91.7)	44 (78.6)	50 (86.2)	-	-	-
Umbilicus	-	-	-	>0.999 ^2^	>0.999 ^2^	>0.999 ^2^
Abnormal	2 (3.3)	1 (1.8)	2 (3.4)	-	-	-
Normal	58 (96.7)	55 (98.2)	56 (96.6)	-	-	-
RUQ	-	-	-	>0.999 ^2^	>0.999 ^2^	>0.999 ^2^
Abnormal	1 (1.7)	0	1 (1.7)	-	-	-
Normal	59 (98.3)	56 (100.0)	57 (98.3)	-	-	-
RLQ	-	-	-	0.260 ^2^	0.677 ^2^	0.486 ^2^
Abnormal	2 (3.3)	5 (8.9)	3 (5.2)	-	-	-
Normal	58 (96.7)	51 (91.1)	55 (94.8)	-	-	-
LUQ	-	-	-	>0.999 ^2^	>0.999 ^2^	>0.999 ^2^
Abnormal	1 (1.7)	1 (1.8)	1 (1.7)	-	-	-
Normal	59 (98.3)	55 (98.2)	57 (98.3)	-	-	-
LLQ	-	-	-	0.028 ^2^	0.059 ^2^	0.775 ^2^
Abnormal	1 (1.7)	7 (12.5)	6 (10.3)	-	-	-
Normal	59 (98.3)	49 (87.5)	52 (89.7)	-	-	-

An abnormal visceral slide test or the presence of abdominal adhesions was defined as less than 1 cm movement of internal organs along the longitudinal axis during forced respiratory inspiration and expiration. ^∗,†,‡^ Comparisons between the two groups with the same symbol. ^1^ Chi-squared or ^2^ Fisher’s exact test was used. Abbreviations: RUQ, right upper quadrant; RLQ, right lower quadrant; LUQ, left upper quadrant; LLQ, left lower quadrant.

**Table 3:** Incidence rate of adhesion symptoms at 4 weeks after surgery.

	Experimental Group ^*, †^	Control Group ^*, ‡^	Comparator Group ^†, ‡^	^*^ *p*	^†^ *p*	^‡^ *p*
Full analysis set	(*n* = 63, %)	(*n* = 57, %)	(*n* = 58, %)	-	-	-
Loss of appetite	-	-	-	0.887 ^1^	0.318 ^1^	0.269 ^1^
0 (none)	57 (90.5)	52 (91.2)	49 (84.5)	-	-	-
1 (mild)	6 (9.5)	5 (8.8)	9 (15.5)	-	-	-
2 (moderate)	0	0	0	-	-	-
3 (severe)	0	0	0	-	-	-
Abdominal pain	-	-	-	0.832 ^1^	0.007 ^1^	0.005 ^1^
0 (none)	51 (81.0)	47 (82.5)	34 (58.6)	-	-	-
1 (mild)	12 (19.0)	10 (17.5)	24 (41.4)	-	-	-
2 (moderate)	0	0	0	-	-	-
3 (severe)	0	0	0	-	-	-
Vomiting	-	-	-	0.497 ^2^	0.497 ^2^	N/A
0 (none)	61 (96.8)	57 (100.0)	58 (100.0)	-	-	-
1 (mild)	2 (3.2)	0	0	-	-	-
2 (moderate)	0	0	0	-	-	-
3 (severe)	0	0	0	-	-	-
Abdominal distention	-	-	-	0.990 ^1^	0.175 ^1^	0.184 ^1^
0 (none)	53 (84.1)	48 (84.2)	43 (74.1)	-	-	-
1 (mild)	10 (15.9)	9 (15.8)	15 (25.9)	-	-	-
2 (moderate)	0	0	0	-	-	-
3 (severe)	0	0	0	-	-	-
Fever higher than 38 °C	-	-	-	N/A	N/A	N/A
0 (none)	63 (100.0)	57 (100.0)	58 (100.0)	-	-	-
1 (mild)	0	0	-	-	-	-
2 (moderate)	0	0	-	-	-	-
3 (severe)	0	0	-	-	-	-
Per protocol set	(*n* = 60, %)	(*n* = 56, %)	(*n* = 58, %)	*-*	*-*	*-*
Loss of appetite	-	-		0.844 ^1^	0.368 ^1^	0.284 ^1^
0 (none)	54 (90.0)	51 (91.1)	49 (84.5)	-	-	-
1 (mild)	6 (10.0)	5 (8.9)	9 (15.5)	-	-	-
2 (moderate)	0	0	0	-	-	-
3 (severe)	0	0	0	-	-	-
Abdominal pain	-	-	-	0.947 ^1^	0.006 ^1^	0.006 ^1^
0 (none)	49 (81.7)	46 (82.1)	34 (58.6)	-	-	-
1 (mild)	11 (18.3)	10 (17.9)	24 (41.4)	-	-	-
2 (moderate)	0	0	0	-	-	-
3 (severe)	0	0	0	-	-	-
Vomiting	-	-	-	>0.999 ^2^	>0.999 ^2^	N/A
0 (none)	59 (98.3)	56 (100.0)	58 (100.0)	-	-	-
1 (mild)	1 (1.7)	0	0	-	-	-
2 (moderate)	0	0	0	-	-	-
3 (severe)	0	0	0	-	-	-
Abdominal distention	-	-	-	0.931 ^1^	0.222 ^1^	0.200 ^1^
0 (none)	50 (83.3)	47 (83.9)	43 (74.1)	-	-	-
1 (mild)	10 (16.7)	9 (16.1)	15 (25.9)	-	-	-
2 (moderate)	0	0	-	-	-	-
3 (severe)	0	0	-	-	-	-
Fever higher than 38 °C	-	-	-	N/A	N/A	N/A
0 (none)	60 (100.0)	56 (100.0)	58 (100.0)	-	-	-
1 (mild)	0	0	0	-	-	-
2 (moderate)	0	0	0	-	-	-
3 (severe)	0	0	0	-	-	-

^∗,†,‡^ Comparisons between the two groups with the same symbol. ^1^ Chi-squared or ^2^ Fisher’s exact test was used. Abbreviations: N/A, not applicable.

**Table 4:** Changes in pain visual analogue score (VAS) scores between baseline and 4 weeks after surgery.

	Experimental Group ^*, †^	Control Group ^*, ‡^	Comparator Group ^†, ‡^	^*^ *p*	^†^ *p*	^‡^ *p*
Full analysis set	(*n* = 63)	(*n* = 57)	(*n* = 58)	-	-	-
Baseline	2.3 ± 7.4 (0.0–38.0)	6.4 ± 11.5 (0–45.0)	5.9 ± 14.0 (0–75.0)	0.007	0.086	0.350
Week 4	3.5 ± 7.5 (0–30.0)	3.6 ± 9.4, (0–50.0)	5.3 ± 9.1 (0–36.0)	0.630	0.150	0.065
Week 4-Baseline	1.2 ± 9.8, (−38.0–30.0)	−2.8 ± 13.2 (−45.0–50.0)	−0.6 ± 16.5 (−75.0–36.0)	0.003	0.383	0.108
Per protocol set	(*n* = 60)	(*n* = 56)	(*n* = 58)	-	-	-
Baseline	1.8 ± 6.1 (0–38.0)	6.5 ± 11.6 (0–45.0)	5.9 ± 14.0 (0–75.0)	0.004	0.060	0.318
Week 4	3.5 ± 7.6 (0–30.0)	3.7 ± 9.5 (0–50.0)	5.3 ± 9.1 (0–36.0)	0.707	0.142	0.073
Week 4-Baseline	1.7 ± 8.7 (−38.0–30.0)	−2.8 ± 13.3 (−45.0–50.0)	−0.6 ± 16.5 (−75.0–36.0)	0.002	0.339	0.104

Presented with mean ± SD (range). ^∗,†,‡^ Comparisons between the two groups with the same symbol. Wilcoxon’s rank sum test was used. Abbreviations: SD, standard deviation.

## Supplementary Materials

The following are available online at https://www.mdpi.com/2077-0383/9/7/2261/s1, Figure S1: Summary of the planned procedures in this clinical trial, Table S1: Overall satisfaction for patients and surgeons at 4 weeks after surgery, Table S2: Surgeon satisfaction with handling anti-adhesion agents on the day of surgery, Table S3: Comparisons of adverse events among the three groups in the safety set.
