# Supplementary material for: A Randomized Controlled Trial of Thermo-Sensitive Sol–Gel Anti-Adhesion Agent after Gynecologic Surgery
Source: J Clin Med. 2020 Jul 16;9(7):2261. doi: 10.3390/jcm9072261 (PMC7408806; doi:10.3390/jcm9072261)
Supplement: Supplementary file 1 [file jcm-09-02261-s001.pdf]

Figure S1. Summary of the planned procedures in this clinical trial.

Period	Screening	Baseline/ Operation	Follow-up	
Visit No.	1	2	3	4
	Within 2 weeks before surgery	Day 0	1 week ± 3 days	4 weeks ± 7 days
Informed Consent	O			
Demographic Survey	O			
Medical History / Drug Administration History	O			
Physical Examination / Vital Signs	O	O	O	O
Laboratory Tests [1]	O		O	O
Pregnancy Test [2]	O			O
Review of Inclusion/Exclusion Criteria	O	O		
Enrollment and Randomisation		O		
Intervention		O		
Evaluation of Efficacy				
- Visceral slide test	O[3]			O
- Assessment of adhesion symptoms			O	O
- Pain assessment (VAS)	O			O
- Surgeon satisfaction assessment		O		O
- Patient satisfaction assessment				O
Medications		O	O	O
Adverse Events		O	O	O

1: Laboratory test results within 4 weeks from the baseline could have been used as the baseline results.
- Hematology: Red blood cells (RBCs), hemoglobin, hematocrit, platelet, white blood cells (WBCs) with differential count (neutrophils, lymphocytes, monocytes, eosinophils, basophils)
- Coagulation test: Prothrombin time (PT), activated partial thromboplastin time (aPTT)
- Biochemistry: Calcium, sodium, potassium, chloride, creatinine, blood urea nitrogen (BUN), alanine aminotransferase (ALT), aspartate aminotransferase (AST), alkaline phosphatase (ALP), total bilirubin, albumin, total protein, total cholesterol, glucose (Fasting), C-reactive protein (CRP)
- Urinalysis: pH, specific gravity, protein, glucose, urobilinogen, ketone, RBCs, WBCs
2: Urine β-HCG pregnancy test was performed at screening and visit 4 for a woman of childbreaing potential.
3: Visceral slide test was performed at screening for evaluation of preoperative abdominal adhesion.

Table S1. Overall satisfaction for patients and surgeons at 4 weeks after surgery

	Experimental group	Comparator group	*p*
Full analysis set	(*n* = 63, %)	(*n* = 58, %)	
**Assessed by patients**			0.875 [1]
0 (poor)	0	0	
1 (fair)	9 (14.3)	6 (10.3)	
2 (good)	12 (19.0)	13 (22.4)	
3 (very good)	19 (30.2)	16 (27.6)	
4 (excellent)	23 (36.5)	23 (39.7)	
Not answered	0	0	
Score	2.9 ± 1.1 (1.0–4.0)	3.0 ± 1.0 (1.0–4.0)	0.716 [2]
**Assessed by surgeons**			<0.001 [1]
0 (poor)	0	0	
1 (fair)	0	30 (51.7)	
2 (good)	1 (1.6)	15 (25.9)	
3 (very good)	32 (50.8)	13 (22.4)	
4 (excellent)	28 (44.4)	0	
Not answered	2 (3.2)	0	
Score	3.4 ± 0.5 (2.0–4.0)	1.7 ± 0.8 (1.0–3.0)	<0.001 [2]
Per protocol set	(*n* = 60, %)	(*n* = 58, %)	
**Assessed by patients**			0.967 [1]
0 (poor)	0	0	
1 (fair)	6 (10.0)	6(10.34)	
2 (good)	12 (20.0)	13(22.41)	
3 (very good)	19 (31.7)	16(27.59)	
4 (excellent)	23 (38.3)	23(39.66)	
Not answered	0	0	
Score	3.0 ± 1.0 (1.0–4.0)	3.0 ± 1.0, (1.0–4.0)	0.955 [2]
**Assessed by surgeons**			<0.001 [1]
0 (poor)	0	0	
1 (fair)	0	30 (51.7)	
2 (good)	1 (1.7)	15 (25.9)	
3 (very good)	31(51.7)	13 (22.4)	
4 (excellent)	28 (46.7)	0	
Not answered	0	0	
Score	3.5 ± 0.5 (2.0–4.0)	1.7 ± 0.8 (1.0–3.0)	<0.001 [2]

Presented with mean ± SD (range) or *n* (%). [1] Chi-squared test and [2] Wilcoxon's rank sum test were used.
Abbreviations: SD, standard deviation.

Table S2. Surgeon satisfaction with handling anti-adhesion agents on the day of surgery

	Experimental group	Comparator group	*p*
Full analysis set	(*n* = 63, %)	(*n* = 58, %)	
**Assessed by surgeons**			<0.001 [1]
0 (poor)	0	0	
1 (fair)	0	30 (51.7)	
2 (good)	0	16 (27.6)	
3 (very good)	32 (50.8)	12 (20.7)	
4 (excellent)	29 (46.0)	0	
Not answered	2 (3.2)	0	
Score	3.5 ± 0.5 (3.0–4.0)	1.7 ± 0.8 (1.0–3.0)	<0.001 [2]
Per protocol set	(*n*= 60, %)	(*n*= 58, %)	
**Assessed by surgeons**			<0.001 [1]
0 (poor)	0	0	
1 (fair)	0	30 (51.7)	
2 (good)	0	16 (27.6)	
3 (very good)	31(51.7)	12 (20.7)	
4 (excellent)	29 (48.3)	0	
Not answered	0	0	
Score	3.5 ± 0.5 (3.0–4.0)	1.7 ± 0.8 (1.0–3.0)	<0.001 [2]

Presented with mean ± SD (range) or *n* (%). [1] Chi-squared test and [2] Wilcoxon's rank sum test were used.
Abbreviations: SD, standard deviation.

Table S3. Comparisons of adverse events among the three groups in safety set

	Experimental group (*n* = 64)			Control group (*n* = 61)			Comparator group (*n* = 58)			***p***
	No. of patients (%) (95% CI) *	No. of events		No. of patients (%) (95% CI) *	No. of events		No. of patients (%) (95% CI) *	No. of events		
**Treatment Emergent Adverse Events (TEAE)**	54 (84.4) (75.5–93.3)	Mild	108	47 (77.0) (66.5–87.6)	Mild	91	50 (86.2) (77.3–95.1)	Mild	105	0.375 [1]
		Moderate	13		Moderate	10		Moderate	14	
		Severe	0		Severe	2		Severe	3	
		Total	121		Total	103		Total	122	
**Adverse Device Effect (ADE)**	1 (1.6) (0.0–8.4)	Mild	1	0 (0.0–5.9)	Mild	0	0 (0.0–6.2)	Mild	0	N/A
		Moderate	0		Moderate	0		Moderate	0	
		Severe	0		Severe	0		Severe	0	
		Total	1		Total	0		Total	0	
**Serious TEAE**	1 (1.6) (0.0–8.4)	Mild	0	1 (1.6) (0.0–8.8)	Mild	0	1 (1.7) (0.0–9.2)	Mild	0	>0.999 [2]
		Moderate	1		Moderate	1		Moderate	1	
		Severe	0		Severe	0		Severe	0	
		Total	1		Total	1		Total	1	
**Serious ADE**	0 (0.0–5.6)	Mild	0	0 (0.0–5.9)	Mild	0	0 (0.0–6.2)	Mild	0	N/A
		Moderate	0		Moderate	0		Moderate	0	
		Severe	0		Severe	0		Severe	0	
		Total	0		Total	0		Total	0	

* 95% confidence interval of incidence rate of TEAE/ADE.

[1] Chi-square or [2] Fisher's exact test was used in comparisons of categorical variables among the three groups.

Abbreviations: CI, confidence interval; N/A, not applicable.
